# Kinetic parameters for nutrient enhanced crude oil biodegradation in intertidal marine sediments

**DOI:** 10.3389/fmicb.2014.00160

**Published:** 2014-04-11

**Authors:** Arvind K. Singh, Angela Sherry, Neil D. Gray, D. Martin Jones, Bernard F. J. Bowler, Ian M. Head

**Affiliations:** ^1^School of Civil Engineering and Geosciences, Newcastle UniversityNewcastle upon Tyne, UK; ^2^Department of Biochemistry, North – Eastern Hill UniversityShillong, Meghalaya, India

**Keywords:** oil spill, bioremediation, kinetics, K_s_, half saturation constant, maximal rates, *Alcanivorax*, *Cycloclasticus*

## Abstract

Availability of inorganic nutrients, particularly nitrogen and phosphorous, is often a primary control on crude oil hydrocarbon degradation in marine systems. Many studies have empirically determined optimum levels of inorganic N and P for stimulation of hydrocarbon degradation. Nevertheless, there is a paucity of information on fundamental kinetic parameters for nutrient enhanced crude oil biodegradation that can be used to model the fate of crude oil in bioremediation programmes that use inorganic nutrient addition to stimulate oil biodegradation. Here we report fundamental kinetic parameters (K_s_ and q_max_) for nitrate- and phosphate-stimulated crude oil biodegradation under nutrient limited conditions and with respect to crude oil, under conditions where N and P are not limiting. In the marine sediments studied, crude oil degradation was limited by both N and P availability. In sediments treated with 12.5 mg/g of oil but with no addition of N and P, hydrocarbon degradation rates, assessed on the basis of CO_2_ production, were 1.10 ± 0.03 μmol CO_2_/g wet sediment/day which were comparable to rates of CO_2_ production in sediments to which no oil was added (1.05 ± 0.27 μmol CO_2_/g wet sediment/day). When inorganic nitrogen was added alone maximum rates of CO_2_ production measured were 4.25 ± 0.91 μmol CO_2_/g wet sediment/day. However, when the same levels of inorganic nitrogen were added in the presence of 0.5% P w/w of oil (1.6 μmol P/g wet sediment) maximum rates of measured CO_2_ production increased more than four-fold to 18.40 ± 1.04 μmol CO_2_/g wet sediment/day. K_s_ and q_max_ estimates for inorganic N (in the form of sodium nitrate) when P was not limiting were 1.99 ± 0.86 μmol/g wet sediment and 16.16 ± 1.28 μmol CO_2_/g wet sediment/day respectively. The corresponding values for P were 63 ± 95 nmol/g wet sediment and 12.05 ± 1.31 μmol CO_2_/g wet sediment/day. The q_max_ values with respect to N and P were not significantly different (*P* < 0.05). When N and P were not limiting K_s_ and q_max_ for crude oil were 4.52 ± 1.51 mg oil/g wet sediment and 16.89 ± 1.25 μmol CO_2_/g wet sediment/day. At concentrations of inorganic N above 45 μmol/g wet sediment inhibition of CO_2_ production from hydrocarbon degradation was evident. Analysis of bacterial 16S rRNA genes indicated that *Alcanivorax* spp. were selected in these marine sediments with increasing inorganic nutrient concentration, whereas *Cycloclasticus* spp. were more prevalent at lower inorganic nutrient concentrations. These data suggest that simple empirical estimates of the proportion of nutrients added relative to crude oil concentrations may not be sufficient to guarantee successful crude oil bioremediation in oxic beach sediments. The data we present also help define the maximum rates and hence timescales required for bioremediation of beach sediments.

## Introduction

Natural hydrocarbon seeps are quantitatively the largest source of petroleum in marine systems, nevertheless, anthropogenic activities involved in the production transport and use of crude oil and oil products remain important sources of oil pollution (National Research Council, 2003). As a result of the localized release of relatively large quantities of oil, anthropogenic emissions may have effects on local ecosystems that are disproportionate to their contribution to global budgets of hydrocarbons in the sea. The incidence of major oil spills has decreased by 76% from 787 to 190 during the four decades from 1970 to 2010. In terms of volume this corresponds to a 93% decrease and, excluding the Deepwater Horizon blowout, the total quantity of oil spilt during 2010–2011 (13,000 tonnes from 13 recorded spills) was the lowest so far recorded (ITOPF, 2011). Although such statistics indicate that oil spills are generally declining, major accidents like the Deepwater Horizon blowout on 20th April 2010 in the Gulf of Mexico are a stark reminder that accidental oil spills remain an important environment hazard. The Deepwater Horizon accident resulted in the world's largest accidental release of crude oil to the sea, releasing an estimated 4.9 million barrels (780,000 m^3^) of light crude oil (OSAT-1, 2010). In offshore regions, the Deepwater Horizon spill had substantial impact on coral communities impacted by the plume from the Macondo well (White et al., 2012). In spite of intensive cleanup efforts, a portion of the spilled Macondo oil drifted to shore and remains trapped in coastal sediments. Concentrations of total petroleum hydrocarbon as high as 510 mg g^−1^ sediment were recorded in the surface 2 cm of heavily polluted marsh sediments even 7 months after the spill (Lin and Mendelssohn, 2012).

Crude oils comprise a complex heterogenous mixture of organic and inorganic compounds and broadly contain four groups of compounds; saturated and aromatic hydrocarbons, resins and asphaltenes (Harayama et al., 1999). Whereas lighter fractions evaporate or are degraded microbially, the heavier and more polar crude oil fractions persist due to their slow degradation rates (Walker et al., 1976). Many hydrocarbon degrading organisms are known (Prince, 2005) and in marine environments a number of specialist hydrocarbon degrading taxa are known (Yakimov et al., 2007). Marine saturated hydrocarbon degrading specialists include *Alcanivorax* (Yakimov et al., 1998), *Planococcus* (Engelhardt et al., 2001), *Oleiphilus* (Golyshin et al., 2002), *Oleispira* (Yakimov et al., 2003), *Thalassolituus* (Yakimov et al., 2004). Aromatic hydrocarbon degraders include *Cycloclasticus* spp. which utilize biphenyl, naphthalene, anthracene, phenanthrene, toluene, and benzoate (Dyksterhouse et al., 1995), and *Neptunomonas* which can degrade naphthalene, 2-methylnaphthalene and phenanthrene as sole carbon sources, but are unable to use 2,6-dimethylnaphthalene, 1-methylnaphthalene, biphenyl or acenaphthene (Hedlund et al., 1999). The chemical complexity of crude oil thus limits the capacity of a single species to degrade only certain components and the combined efforts of mixed bacterial consortia improve hydrocarbon bioremediation in marine environments (Röling et al., 2002; Dell'Anno et al., 2012). However, artificial microbial consortia cannot substitute for highly complex and dynamic indigenous microbial population essential for complete and efficient hydrocarbon degradation (McKew et al., 2007a).

Marine bacteria from the genera *Alcanivorax* and *Cycloclasticus*, have been implicated as key hydrocarbonoclastic agents on a global scale (Maruyama et al., 2003; Cappello et al., 2007). Their abundances and hydrocarbon degradation activity in polluted environments often increases significantly with a concomitant reduction in overall bacterial diversity (MacNaughton et al., 1999; Kasai et al., 2001, 2002a,b; Röling et al., 2002; Cappello et al., 2007; McKew et al., 2007b). A study on bacterial community response in beach sediment impacted by the Deepwater Horizon oil spill demonstrated that *Alcanivorax* spp. became dominant in polluted sediments and responded rapidly in the early stages following oiling (Kostka et al., 2011; Newton et al., 2013).

A 16S rRNA gene, PCR based denaturing gradient gel electrophoresis (DGGE) analysis and qPCR analysis of microbial population in nutrient amended crude oil treated marine sediment plots revealed an increase in number of *Alcanivorax* spp. and simultaneous appearance of *alkB* genes coding for alkane hydroxylase responsible for catabolism of alkanes (Röling et al., 2004; Singh et al., 2011). The success of *Alcanivorax* spp. as alkane degraders in part lies in their ability to use both branched chain and straight chain alkanes efficiently as sources of carbon and energy (Hara et al., 2003). Importantly, although *Alcanivorax borkumensis* SK2 genome has been shown to possess high affinity permeases for nitrate and phosphorus (Schneiker et al., 2006) it has been shown that the nitrate transporter *ntr*B gene and *nir*B1 for nitrite reductase are down-regulated in the presence of hexadecane by 3.93- and 6.5-fold respectively (Sabirova et al., 2011). Aromatic hydrocarbon degraders also exhibit a strong positive response to nutrient amendments. Abundance of *Cycloclasticus* spp. in heat treated Arabian light crude oil polluted gravel was shown to increase by 5 orders of magnitude under inorganic nutrient treated conditions and by 2 orders of magnitude under oil contaminated conditions with no nutrients, relative to unoiled sediments without nutrient amendments (Kasai et al., 2002b).

Since *Alcanivorax* spp and *Cycloclasticus* spp. do not compete for organic compounds as carbon sources, their initial abundance, metabolic superiority, and growth rate can be very crucial for determining their emergence, activity and ultimate relative abundance in hydrocarbon polluted environments. While these taxa do not compete directly for carbon and energy sources in oil-polluted environments they do compete for electron acceptors and inorganic nutrients and this may dictate the relative degradation of saturated and aromatic hydrocarbons. Indeed nutrient supply has been shown to have differential effects on rates of aliphatic and aromatic hydrocarbon degradation which has been interpreted in the context of resource ratio theory (Smith et al., 1998). Moreover, there is some evidence that inorganic nutrient availability controls selection of different *Alcanivorax* genotypes (Röling et al., 2002; Head et al., 2006).

Biostimulation efficiently enhances hydrocarbon bioremediation activity (McKew et al., 2007b) and typically saturated hydrocarbon degradation is stimulated initially followed by degradation of aromatic hydrocarbons and polar components respectively (Fusey and Oudot, 1984). In some instances losses of aromatic hydrocarbons before saturated hydrocarbons have been observed (Jones et al., 1983; Cooney et al., 1985). Such differences in hydrocarbon removal patterns could be due to relative growth efficiency of aromatic and aliphatic hydrocarbon degrading organisms under prevailing environmental conditions and their initial abundance. The goal of hydrocarbon bioremediation strategies is to allow degradation activity at maximum rates by providing nutrients in quantities sufficient to support the growth of hydrocarbon degrading organisms and microbial hydrocarbon degradation activity was shown to increase up to 2.5 mg N/L (0.18 mM) beyond which nutrient level does not enhance the rate of degradation (Boufadel et al., 1999). A continuous supply of inorganic nutrient in combination with sand amendments for efficient mass transfer also has been shown to enhance kinetics of microbial growth, and hydrocarbon degradation (Beolchini et al., 2010).

Although biostimulation of hydrocarbon degradation processes has been studied extensively, there has been very limited attempt to systematically understand the kinetics of nutrient enhanced biodegradation of crude oil and to correlate this with the emergence of specific microbial population in hydrocarbon contaminated marine sediments (Röling et al., 2004; Beolchini et al., 2010). The present study therefore focusses on estimation of kinetic parameters for inorganic nutrient-enhanced hydrocarbon degradation and their effect on the microorganisms responsible.

## Materials and methods

### Sample collection and microcosm set up

Beach sediment samples consisting of fine sand were collected on 6/11/2009 in sterilized glass bottles (Duran) from a site close to St Mary's Island near Whitley Bay, Newcastle upon Tyne, United Kingdom (N 55°04′ 18″, W 01°26′ 59″). Sediment samples were stored at 4°C for a maximum 24 h prior to the start of the experiments. Oil degrading microcosms comprising beach sediment (10 g), North Sea crude oil (125 mg) and different concentrations of inorganic nutrients (sodium nitrate and potassium dihydrogen phosphate) were prepared in triplicate in serum bottles (114 ml capacity). The oil was weighed directly into the serum bottles, the sediment was added and the nutrient solution was pipetted onto the sediment to give the appropriate levels of nutrients (see “Effect of inorganic nutrient concentration” below). The total volume of nutrient solution added was always made up to 250 μl so that all serum bottles received the same amount of liquid. The sediment, oil and nutrient solution were mixed gently with a glass rod and the microcosms were sealed with butyl rubber stoppers and incubated at 24°C in darkness. Microcosms without nutrient amendment, amended with 250 μl of water served as a control. We monitored oxygen content in the headspace simultaneously with CO_2_ by GC-MS (see below) and in the 6 day incubation period the headspace remained oxic. Our measurements showed that by day 6 the degree of oxygen depletion was 70.1 ± 0.1% (*n* = 24) of the initial levels. In long term incubations where greater oxygen consumption occurred with increasing oil degradation, the headspace was replaced with air when oxygen dropped below 15% by volume.

### Effect of inorganic nutrient concentration on crude oil degradation

Inorganic nutrient treatments were nitrogen alone (0–5% w/w oil), different levels of phosphorus with a constant inorganic nitrogen concentration (0–0.5% P and 3% N w/w oil), or different levels of inorganic nitrogen with constant phosphorus concentration (0–5% N with 0.5% P w/w of oil) all treatments were conducted in triplicate. Control incubations with nutrients and no added oil were also conducted to determine the contribution of indigenous organic carbon to CO_2_ production.

### Effect of crude oil concentration on oil degradation

Microcosms set up as described above were prepared in triplicate with different amounts of oil ranging from 10 to 500 mg of crude oil and 6.25 mg N (44.6 μmole/g sediment) and 0.625 mg P (2.02 μmole/g sediment). This gives a range of N and P levels ranging from 62.5% N and 6.25% P w/w of oil with 10 mg of oil to 1.25% N and 0.125% P w/w of oil with 500 mg of oil. With 125 mg of oil this is equivalent to 5% N and 0.5% P w/w of oil. The effect of crude oil levels on oil biodegradation was also investigated by treatment with different quantities of crude oil (10–500 mg) but a constant ratio of 5% N and 0.5% P w/w of oil. This was conducted because bioremediation treatments often recommend that a particular mass of inorganic nutrients is supplied relative to the amount of oil present (Swannell et al., 1996). In these treatments the absolute concentration of inorganic nutrients therefore increases with the amount of crude oil present Thus, experiments treated with a single level of nutrients contained approximately 45 μmol N/g wet sediment, while those that contained a constant ratio of inorganic nutrients relative to the mass of oil had N concentrations ranging from around 3.5–180 μmol N/g wet sediment. If the same amount of nutrient added to the 500 mg oil treatment was added to 125 mg of oil (as used in all other microcosms) this would equate to 20% N and 2% P w/w of oil.

### Estimation of kinetic parameters

Rate data in response to different inorganic nutrient and oil concentrations were fitted to a Monod-type kinetic model (q = q_max_^*^[S]/K_s_ + [S]) using non-linear regression implemented in SPSS (IBM SPSS Statistics 19.0.0.1). This was used to derive the model parameters, K_s_ (half saturation constant) and q_max_ (maximal rates).

### Carbon dioxide measurement

Carbon dioxide production as a measure of microbial activity and crude oil degradation was assessed in microcosm headspace samples daily over a period of 6 days using GC-MS. Maximal rates of CO_2_ production were calculated from the steepest part of the CO_2_ accumulation curve which typically followed a lag of 3–4 days (Figure S1). Analysis was performed on a Fisons 8060 GC linked to a Fisons MD 800 MS (electron voltage 70 eV, source temperature 200°C, interface temperature 150°C). Hundred micro liter of headspace gas was manually injected via a syringe (SGE Analytical Science) under an atmosphere of N_2_ gas. Injection through a manifold which is continuously flushed with N_2_ was used to prevent interference from any ingress of air from the atmosphere during injection. The sample was separated using a HP-PLOT-Q capillary column (30 m × 0.32 mm). Helium was used as the carrier gas (1 ml/min, 65 kPa, split at 100 ml/min; 250°C). Data acquisition, integration and quantification were controlled using Xcalibur 1.2 software. A mixture of gases with 10% CO_2_ was used as a standard for calibration. Different volumes of standard gas mix were used to produce a calibration curve which was linear over the range of 1–10% CO_2_. Percent CO_2_ values were converted to total molar masses for determination of cumulative CO_2_ production. *R*^2^ values for calibration curves ranged from 0.993 to 0.997.

### Residual oil extraction and analysis

Petroleum hydrocarbons from the North Sea crude oil treated microcosm sediments were extracted using a mixture of dichloromethane (DCM):methanol (93:7). Prior to extraction a known quantity of squalane was added in to the sediment as surrogate extraction standard. DCM:methanol (20 ml) was added to the sediment in serum bottle microcosms and stored at room temperature overnight. Microcosms were then sonicated for 1 min and the resulting supernatant was transferred into a flask with this extraction procedure being repeated twice more. The solvent containing the extract was passed through an alumina short column (1 cm bed depth) and then rotary evaporated to dryness before being redissolved in DCM. An aliquot of the organic extract in DCM was evaporated to dryness using a stream of nitrogen gas and solvent exchanged into hexane (200 μl). The total solution was added to a 500 mg/3 ml capacity Isolute^®^ C-18 Solid Phase Extraction (SPE) column prewashed with hexane, and eluted with hexane (5 ml). The eluate was transferred into an autosampler vial and made up to 1 ml with hexane, together with a known amount of heptadecylcyclohexane internal standard. This saturated hydrocarbon fraction was analyzed using an Agilent (HP) 5890 Series II gas chromatograph (GC) fitted with a flame-ionization detector (FID). Samples were injected via split-splitless injector (held at 300°C) using an autosampler. The GC was fitted with a 30 × 0.25 mm fused silica capillary column coated with HP-5 phase (0.25 μm). Hydrogen was used as the carrier gas at a flow rate of 2 ml/min. An initial oven temperature of 50°C was held for 2 min and was then heated to 300°C at 5°C/min., where it was held for 20 min. Data were acquired and processed using Thermo LabSystems Atlas software.

### DNA extraction

DNA from 500 mg of frozen microcosm sediment was extracted using a FastDNA^®^ SPIN Kit for Soil (MP Biomedicals™) and a ribolyser (Thermo) according to the manufacturer's instruction. The DNA was eluted in sterilized milliQ water (50 μl) and frozen at −20°C prior to further analysis. The remaining 9.5 g of sediment was used for hydrocarbon extraction and analysis.

### Primer design

Primers for amplifying 16S rRNA gene fragments from the total bacterial population and *Alcanivorax* spp. were designed using the probe and PCR primer design software tool Primrose (Ashelford et al., 2002) (Table 1). For *Alcanivorax*, the forward primer A16SF.493 matched 1606 of 2004048 bacterial sequences in the RDP database release 11, including 1059 of 1108 *Alcanivorax* sequences. The reverse primer A16SR.659 matched 1144 of 2004048 bacterial sequences in the RDP release 11 including 1058 of 1108 *Alcanivorax* sequences. The two primers in combination target 1020 of 2004048 bacterial sequences including 1016 of the 1108 *Alcanivorax* spp. 16S rRNA gene sequences in the database. The inosine-containing primer pair for total bacterial 16S rRNA genes (Gray et al., 2011; Callbeck et al., 2013) targets 855621 of 944469 bacterial sequences with the relevant target region in the RDP database.

**Table 1:** **Oligonucleotides primers used in qPCR analysis**.

**Primer set**	**Sequence (5′–3′)**	**Target organisms**	**Primer location**
Al 6S F.493	CACCGGCTAATTTCGTGC	*Alcanivorax*	481–498^*^
Al 6S R.659	ACCGGAAATTCCACCTCC	*Alcanivorax*	647–664^*^
U 1048f	GTGITGCAIGGIIGTCGTCA	*Bacteria*	1048–1068^**^
U1371	ACGTCITCCICICCTTCCTC	*Bacteria*	1352–1371^**^

* Site on 16S rRNA gene of Alcanivorax borkumensis SK2 (S000018396);

** Site on E.coli 16S rRNA gene.

### PCR-amplification of 16S rRNA genes

Near full length 16S rRNA gene fragments were amplified using primer pair pA and pH (Edward et al., 1988) as described in Röling et al. (2004). For DGGE analysis, 16S rRNA gene fragments were amplified using primers 2 and 3 (Muyzer et al., 1993) as described previously (Röling et al., 2004). All PCR reactions were conducted using a PC Gene thermal cycler.

### Agarose gel and denaturing gradient gel electrophoresis (DGGE)

Agarose gel electrophoresis of PCR-amplifed 16S rRNA gene fragments from *Alcanivorax* spp. was run for 45 min at 80V using a 1.5% (w/v) agarose gel in 1 x TAE buffer. DGGE was conducted at 60°C using a 0.75 mm thick 10% polyacrylamide gel (ratio of acrylamide to bisacrylamide, 37.5:1) with a concentration gradient of 30–55% of denaturant using a Bio-Rad Dcode system. Gels were stained and photographed according to Röling et al. (2004). 100% denaturant comprised 7M Urea and 40% (vol/vol) deionized formamide in TAE buffer. 1 X TAE buffer contained 40 mM Tris-acetate, 1 mM EDTA, pH 8.0). Cloned *Alcanivorax* sp. 16S rRNA genes amplified using primers 2 and 3 (Muyzer et al., 1993) were used as markers for qualitatively identifying DGGE bands related to *Alcanivorax* spp. in DGGE profiles of total bacterial population 16S rRNA genes.

### 16S rRNA gene cloning

DNA extracted from beach sediment as described above, was used to prepare a bacterial 16S rRNA gene clone library from a beach microcosm containing crude oil and treated with 1% N and 0.1% P after 5 days of incubation. The PCR-amplified 16S rRNA gene fragments were cloned with a TOPO^®^ cloning kit (Invitrogen) as per the manufacturer's instructions. The clone libraries were screened for *Alcanivorax* sp 16S rRNA genes using the primer pairs listed in Table 1. Cloned *Alcanivorax* 16S rRNA genes were used to prepare standards for qPCR. These primer pairs were also used for detecting the presence of *Alcanivorax* spp. in microcosm sediments treated with different levels of inorganic nutrients.

### Quantitative PCR

Quantitative PCR (qPCR) was used to determine the abundance of bacterial 16S rRNA genes using primer pair U1048f and U1371 (Gray et al., 2011; Callbeck et al., 2013), and abundance of *Alcanivorax* 16S rRNA genes was quantified using primer pair A16SF.493 and A16SR.659 (Table 1). qPCR was performed in 20 μl of reaction mixture using an iCycler (iQ™5 multicolor, Bio-Rad, Hemel Hempstead, UK) as described in Singh et al. (2011) with the following temperature cycles: one cycle of initial denaturation at 95°C for 7 min followed by 40 cycles of 95°C for 30 s, 61°C for 60 s and 72°C for 40 s. A standard curve for qPCR was prepared by dilution of a PCR-amplified cloned *Alcanivorax* 16S rRNA gene fragment. Standard curves had *R*^2^ values greater than 0.97 and calculated amplification efficiencies ranged from 101 to 113%. The PCR-amplified 16S rRNA gene was gel purified using QIAquick PCR purification kit (Qiagen) and quantified using a nanodrop^®^ ND-1000 spectrophotometer. The number of 16S rRNA gene copies in the undiluted sample was calculated using the formula described by McKew et al. (2007b) and used to prepare a dilution series ranging from 10^8^ to 10^0^ target genes per μl.

### Statistical analysis

Two sample *t*-tests assuming unequal variances, and single factor ANOVA were performed using Microsoft Excel and non-linear regression for estimation of kinetic parameters was conducted using IBM SPSS Statistics 19.0.0.1.

## Results

### Effect of inorganic nutrient concentration on crude oil degradation

**Figure 1:**
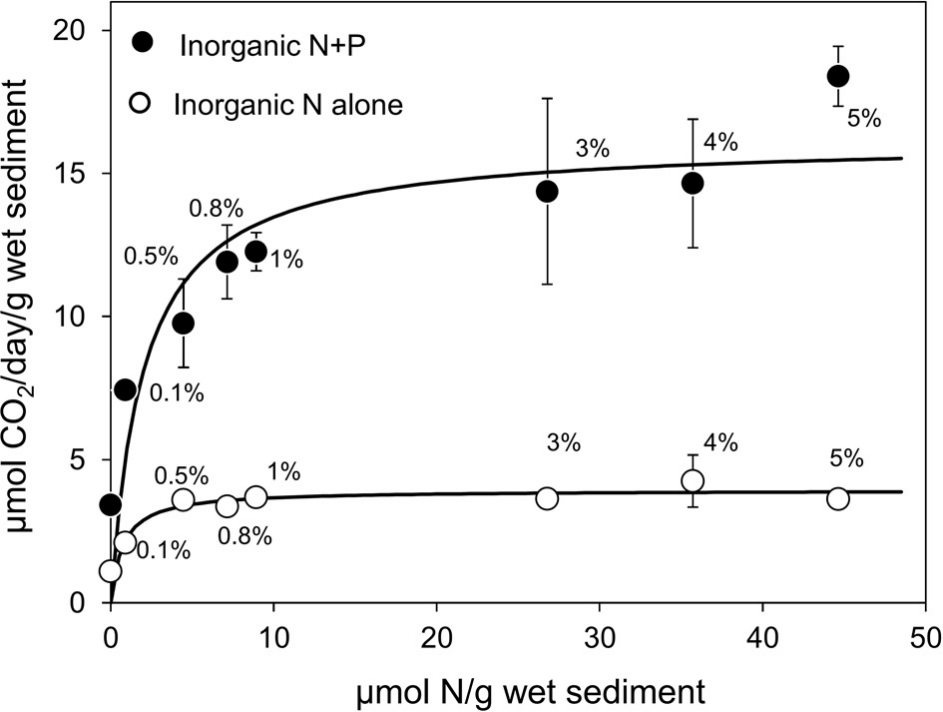
**Effect of inorganic N treatment alone (0–5% N w/w of oil—open circles) or inorganic N (0–5% w/w of oil) with constant P (0.5% w/w of oil) treatment (filled circles) on the rate of CO_2_ production in microcosms containing 10 g beach sediments and 125 mg North Sea crude oil**. Data are plotted as micromoles N/g sediment with the equivalent %N w/w of oil annotated next to each data point. Sodium nitrate and potassium dihydrogen phosphate were used as N and P sources. Each data point represents the average value of three replicates. Where error bars are not seen they are smaller than the symbols. Control incubations with nutrients and no added oil were also conducted to determine the contribution of indigenous organic carbon to CO2 production. These typically gave values of 1.05 ± 0.27 μmol CO2/g wet sediment/day (see Figure S1).

Microcosms treated with N but without P amendment exhibited stimulation of microbial activity. The activity at 0% N and 0% P (1.18 ± 0.15 μmol CO_2_ produced/g wet sediment/day) was not significantly different from rates of CO_2_ production in sediments treated with no added oil (1.05 ± 0.27 μmol CO_2_/g wet sediment/day). Activity when inorganic N alone and oil were present was significantly higher. At 0.1% N and 0% P the rate of CO_2_ production was 2.01 ± 0.06 μmol CO_2_/g wet sediment/day (*t*-test; *P* = 0.0095) or with 0.5% N and 0% P 3.66 ± 0.05 μmol CO_2_/g wet sediment/day (*t*-test; *P* = 0.000041). Without P treatment, measured oil degrading activity reached a maximum level at 4% N w/w of oil (4.25 ± 0.91 μmol CO_2_ produced/g wet sediment/day) and at levels of N of 0.5% w/w of oil and above there was no significant difference in the rate of crude oil biodegradation (ANOVA; *P* = 0.750; Figure 1). These data indicated that crude oil degradation was nitrogen limited, but at levels of nitrogen 0.5% w/w of oil and above another factor became limiting.

Microcosms treated with both inorganic N and P showed an enhancement of oil degradation over and above that seen with nitrogen alone (Figure 1). For example CO_2_ production in 0.5% N and 0.5% P treated microcosms (9.77 ± 1.54 μmol CO_2_ produced/g wet sediment/day) was significantly higher than the activity observed with 0.5% N and 0% P (3.66 ± 0.05 μmol CO_2_ produced/g wet sediment/day) (*t*-test; *P* = 0.029). Activity ranged from 3.41 ± 0.25 μmol CO_2_ produced/g wet sediment/day at 0% N/0.5% P concentration to 18.40 ± 1.04 μmol CO_2_ produced/g wet sediment/day at 5% N/0.5% P but no significant stimulation of oil degradation was seen at N levels greater than 0.5% w/w of oil when P was not limiting (ANOVA; *P* = 0.207, Figure 1).

These data were used to estimate half saturation constants and maximal rates using non-linear regression to a Monod-type kinetic model (q = q_max_ × [S]/K_s_ + [S]). In sediments with no added P, K_s_ and q_max_ for inorganic nitrogen was 0.72 ± 0.32 μmol N/g sediment and 3.93 ± 0.22 μmol CO_2_ produced/g wet sediment/day. The K_s_ and q_max_ for hydrocarbon degradation activity when P was not limiting were 1.99 ± 0.87 μmol N/g wet sediment and 16.16 ± 1.28 μmol CO_2_/g wet sediment/day respectively (Figure 1). The K_s_ values for N, with and without P addition, were not statistically significantly different (*P* > 0.05) whereas q_max_ when both N and P were provided was significantly (over four times) greater than q_max_ when only N was provided (*P* < 0.05).

**Figure 2:**
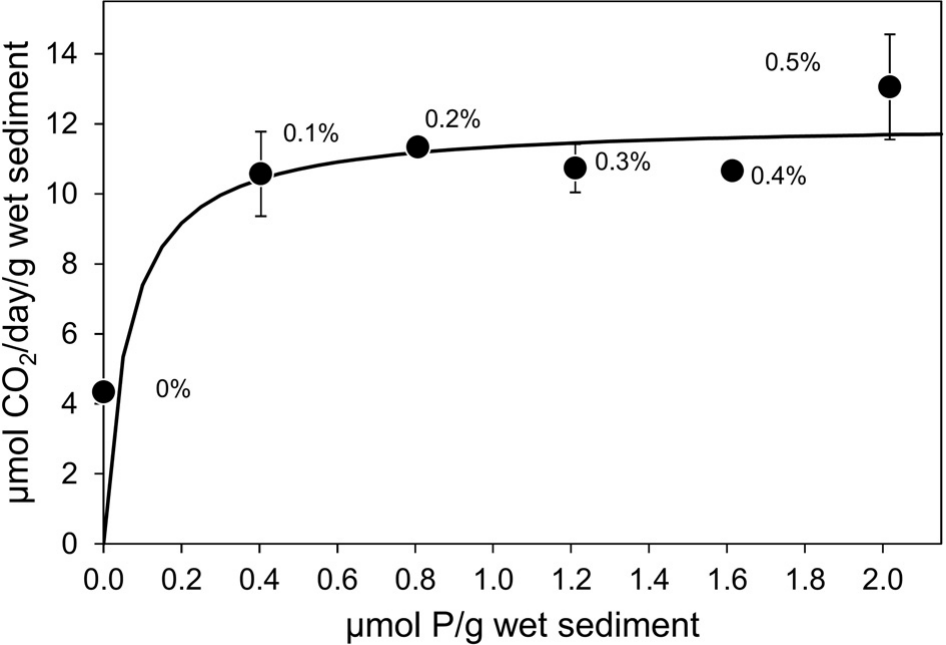
**Effect of inorganic phosphorus (0–0.5% P w/w of oil) with constant nitrogen concentration (3% N w/w of oil) on the rate of CO_2_ production in microcosms containing 10 g beach sediments and 125 mg North Sea crude oil**. Data are plotted as micromoles P/g sediment with the equivalent %P w/w of oil annotated next to each data point. Sodium nitrate and potassium dihydrogen phosphate were used as N and P sources. Each data point represents the average value of three replicates. Where error bars are not seen they are smaller than the symbols.

To systematically determine the level at which P became limiting, microcosms amended with 3% N w/w of oils and different P concentrations were analyzed. With 3% N and 0% P the oil degradation rate was 3.63 ± 0.28 μmol CO_2_ produced/g wet sediment/day. This was significantly lower (*P* = 0.04) than rates of CO_2_ production with 3% N and 0.1% P (14.37 ± 3.24 μmol CO_2_ produced/g wet sediment/day; Figure 2). With P ranging from 0.1 to 0.5% rates of CO_2_ production ranged from 10.56 ± 1.21 μmol CO_2_ produced/g wet sediment/day to 13.05 ± 1.51 μmol CO_2_ produced/g wet sediment/day and there was no significant difference between the 0.1 and 0.5% P treatments (ANOVA; *P* = 0.340).

It was thus clear that P limitation of oil degradation was alleviated above 0.1% P w/w of oil. The K_s_ and q_max_ values estimated for P were 63 ± 95 nmol/g wet sediment and 12.05 ± 1.31 μmol CO_2_/g wet sediment/day. The q_max_ values determined with respect to N (Figure 1) and P (Figure 2) were not statistically significantly different (*P* > 0.05).

### Effect of oil concentration on crude oil biodegradation

Effect of crude oil concentration ranging from 1 mg/g wet sediment to 50 mg/g wet sediment on oil degrading microbial activity was investigated. The microcosms were amended either with a single level of inorganic nutrients irrespective of the amount of oil added (0.625 mg N and 0.0625 mg of P per gram of sediment (equivalent to 5% N and 0.5% P w/w of 125 mg of crude oil) or with a constant ratio of 5% N and 0.5% P w/w of crude oil leading to a range of nutrient levels ranging from 0.05 mg N/0.005 mg P to 2.5 mg N/0.25 mg P per gram of sediment equivalent to 20% N/2% P if in total 125 mg of oil rather than 500 mg of oil was present in the microcosms.

**Figure 3:**
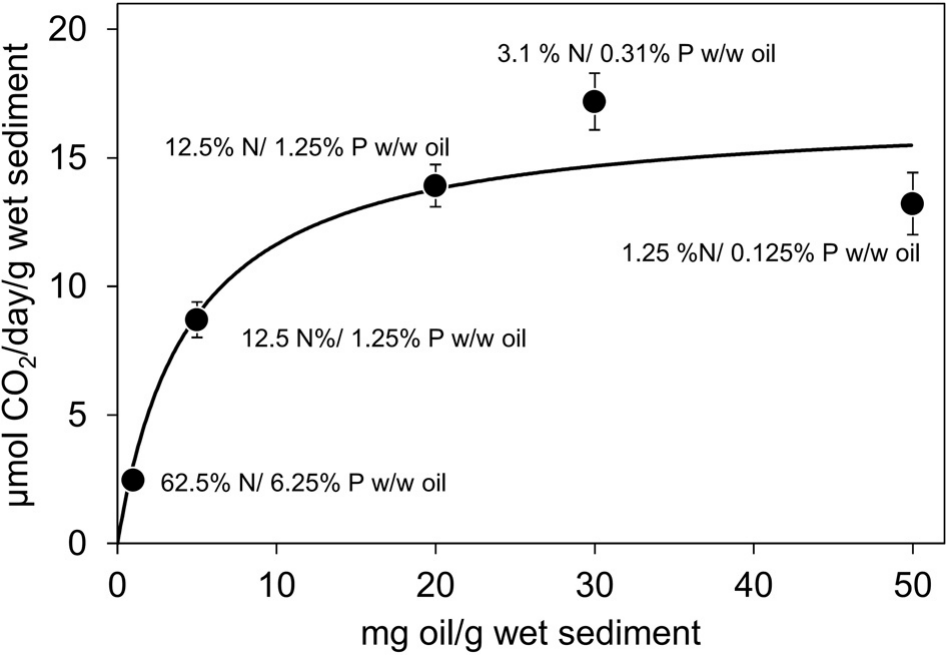
**Effect of oil concentration on the rate of CO_2_ production in microcosm comprising 10 g beach sediments, 6.25 mg N (44.6 μmole/g sediment) and 0.625 mg P (2.02 μmole/g sediment) and 10–500 mg of North Sea crude oil**. This gives a range of N and P levels ranging from 62.5% N and 6.25% P w/w of oil with 10 mg of oil to 1.25% N and 0.125% P w/w of oil with 500 mg of oil. With 125 mg of oil this is equivalent to 5% N and 0.5% P w/w of oil. Sodium nitrate and potassium dihydrogen phosphate were used as N and P sources. Each data point represents the average value of three replicates. Where error bars are not seen they are smaller than the symbols.

Where a single level of inorganic nutrients was provided, the rate of CO_2_ evolution increased with increasing quantity of oil between 1 and 20 mg/g sediment (*t*-test: *P* = 0.000003; Figure 3). At crude oil concentrations ranging from 20 to 50 mg/g sediment there was no significant difference in the rate of CO_2_ production with increasing oil concentration (ANOVA: *P* = 0.08; Figure 3). Thus, up to these levels, equivalent to 5% oil by weight of sediment, crude oil was not auto-inhibitory.

**Figure 4:**
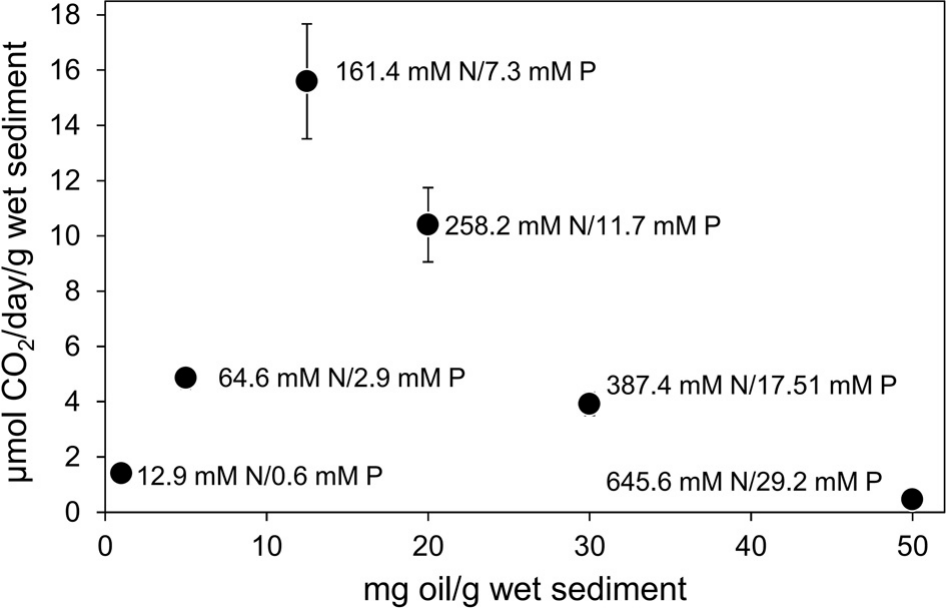
**Effect of oil concentration on the rate of CO_2_ production in microcosm comprising 10 g beach sediment and 10–500 mg North Sea crude oil amended with a constant ratio of 5% N and 0.5% P w/w of oil**. Sodium nitrate and potassium dihydrogen phosphate were used as N and P sources. Each point is annotated with the concentration of N and P in millimolar terms, based on the water content of the sediments. Each data point represents the average value of three replicates. Where error bars are not seen they are smaller than the symbols.

**Figure 5:**
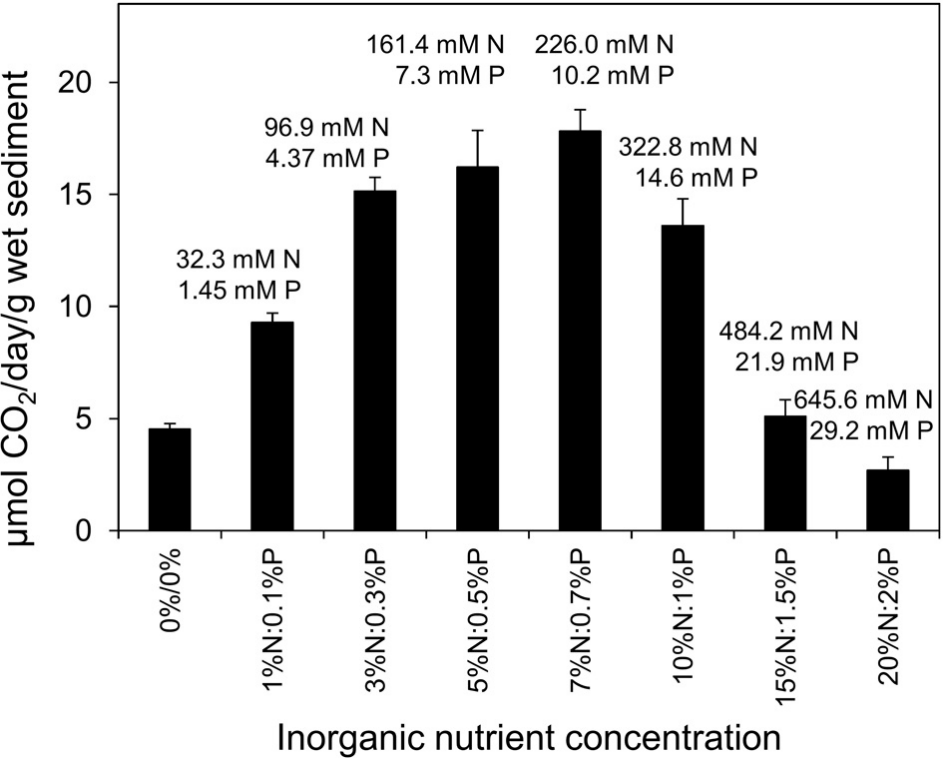
**Effect of inorganic N treatment (0–20% w/w of oil) with 1/10^th^ P treatment on the rate of CO_2_ production in microcosm comprising 10 g beach sediment and 125 mg North Sea crude oil**. Sodium nitrate and potassium dihydrogen phosphate were used as N and P sources. For easy cross referencing to Figure 4, each point is annotated with the concentration of N and P in millimolar terms, based on the water content of the sediments. Each data point represents average value of three replicates. One percent N by weight of oil is equivalent to approximately 9 micromoles of N per g sediment and 20% N is equivalent to 178 micromoles of N per g sediment.

When a constant *ratio* of inorganic nutrients was provided with increasing oil concentration, CO_2_ production rate decreased when oil levels were greater than 12.5 mg oil/g sediment (Figure 4). The CO_2_ production rate at 20 mg oil/g sediment (10.40 ± 1.35 μmol CO_2_ produced/g wet sediment/day) was less than the rate at 12.5 mg oil/g sediment (15.59 ± 2.08 μmol CO_2_ produced/g wet sediment/day) and with 50 mg oil/g sediment the rates dropped further to 0.46 ± 0.04 μmol CO_2_ produced/g wet sediment/day, equivalent to almost a 97% decrease compared to rate at 12.5 mg oil/g sediment. Differences in CO_2_ production rates with 12.5 mg oil/g sediment and higher oil concentrations were statistically significant (ANOVA: *P* = 0.00012). The inhibition of oil degrading activity at a lower oil concentration than that seen when inorganic nutrients were added at a single concentration was most likely due to toxicity of the higher absolute amounts of nutrients present in microcosms containing higher levels of oil. If the microcosm containing 50 mg of oil per gram of sediment is considered, the level of nutrients applied would be equivalent to 20% N and 2% P w/w of oil in a treatment containing 12.5 mg oil/g sediment. Indeed a systematic evaluation of the effect of nutrient concentration ranging from 0 to 20% N with 1/10th the P concentration w/w of 125 mg of oil showed that nutrient levels greater than 7% N/ 0.7% P w/w of oil (equivalent to 62.5 micromoles N and 2.83 micromoles P/g sediment or 226 mM N/10.2 mM P) resulted in a reduction in CO_2_ production rate from oil degradation (Figure 5).

### Alkane degradation in microcosm incubations

At the end of the 6 day incubation period residual crude oil was extracted from the microcosms treated with 125 mg crude oil and a range of inorganic nutrient concentrations (0%N/0.5%P to 5%N/0.5%P w/w of oil). The saturated hydrocarbon fractions were isolated and the resolved *n*-alkanes, pristane and phytane were quantified. The *n*C_12_ to *n*C_32_ alkanes present in the oil comprise approximately 10% by weight of the oil and thus the amount of resolved alkanes at the start of the experiment was about 12,500 μg per microcosm in addition volatile hydrocarbons (*n*C_5_ to *n*C_10_ and benzene and toluene) comprise around 7000 μg per microcosm. Across all treatments the total amount of alkanes measured (the sum of *n*C_12_–*n*C_32_) ranged from 9833 ± 1623 to 12168 ± 628 μg per microcosm. This suggests that a moderate amount of alkane degradation occurred over the 6 day incubation. Indeed, there was no statistically significant difference in the total mass of alkanes recovered, irrespective of the inorganic nutrient amendment (ANOVA: *P* = 0.244). This suggested that the degree of hydrocarbon degradation had been moderate. In addition to losses due to biodegradation some lower molecular weight alkanes may have been lost due to evaporation. Even though the incubations were conducted in sealed serum bottles some evaporative losses may have occurred during sampling the headspace for CO_2_. A systematic analysis of evaporative loss of volatile alkanes in the headspace demonstrated that flushing the headspace with up to 1800 ml of air removed alkanes up to *n*C_9_ to varying degrees, but *n*C_10_ was unaffected (Figure S2). Alkanes with lower molecular weight than *n*C_12_ were lost during the procedure for purification of the saturated hydrocarbon fraction and are not accounted for in our figures.

**Figure 6:**
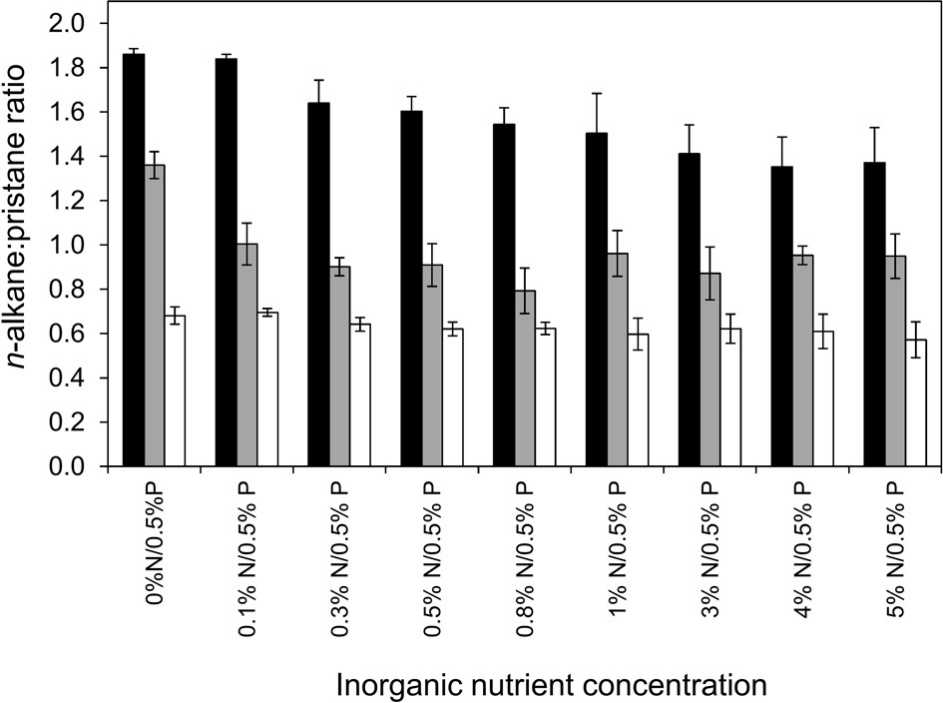
**Effect of inorganic N (0–5%) with constant P (0.5%) treatment w/w of oil on the degradation on n-alkane (nC17, nC13, and nC25) to pristane ratios in microcosm comprising 10 g beach sediment and 125 mg North Sea crude oil**. Each data point represents average value of three replicates. nC17:pristane black bars, nC13:pristane gray bars, nC25:pristane white bars.

A more sensitive way to determine *n*-alkane degradation is by measuring the ratio of *n*-alkanes (typically *n*C_17_) relative to the concentration of the more slowly degraded branched alkane, pristane. To assess the degree of degradation of alkanes of different molecular weight we determined *n*C_13_:pristane, *n*C_17_:pristane and *n*C_25_:pristane ratios for all of the treatments (Figure 6).

The nC_17_:pristane ratio of the starting oil was 1.99 ± 0.01. The range of *n*C_17_:pristane ratios across treatments was relatively small with a maximum value in the 0%N/0.05%P treatment of 1.86 ± 0.04 and the lowest value measured in the 4%N/0.5%P treatment (1.35 ± 0.23). There were significant differences in the *n*C_17_:pristane ratios across all treatments (ANOVA: *P* = 0.036) resulting from lower values measured in treatments from 0.3% N/0.5%P to 5%N/0.5% (1.35 ± 0.23 to 1.64 ± 0.18) compared to the 0%N/0.5%P and 0.1%N/0.5%P treatments (1.86 ± 0.04 and 1.84 ± 0.04). There were no significant differences in the *n*C_17_:pristane ratios between the 0.3%N/0.5%P to 5%N/0.5% treatments (ANOVA: *P* = 0.589). If *n*C_18_:phytane ratios were used the results were essentially the same as obtained with *n*C_17_:pristane ratios. A similar pattern was seen with *n*C_13_:pristane ratios except that all nutrient treatments greater than 0%N/0.5%P gave *n*C_13_:pristane ratios which were statistically indistinguishable (ANOVA: *P* = 0.815) but were significantly different from the 0%N/0.5%P treatment (*P* = 0.018). There was no difference in *n*C_25_:pristane ratios across all treatments (ANOVA: *P* = 0.834). Taken together these data indicate that there was a small degree of degradation of *n-*alkanes and that lower molecular weight alkanes were degraded to a greater degree than higher molecular weight alkanes over the short 6 day time course of the experiments (Figure 6). Estimation of the extent of oil degradation based on a mass balance from the CO_2_ produced over the 6 day incubation period, indicated that the CO_2_ generated could account for degradation of 6.19 ± 0.02 to 37.28 ± 2.06% of the total mass of *n*C_5_–*n*C_32_ alkanes and volatile low molecular weight aromatics (benzene and toluene) initially present, in the 0%N/0.5%P and 5%N/0.5%P treatments respectively. Estimates based on the *n*C_17_:pristane ratio were generally similar and ranged from 6.49 to 32.08%. Notwithstanding differences in the volatility of *n*C_13_ and pristane, estimates of degradation based on the *n*C_13_:pristane ratio indicated a greater degree of degradation with a maximum estimated extent of degradation of 49.85%. Discrepancies in these estimates likely reflect the fact that the CO_2_ produced integrates degradation of all components of the oil that are being removed whereas the alkane:pristane ratio data provide information on selected compounds which, as a comparison of the *n*C_13_:pristane and *n*C_17_:pristane suggests, are degraded to different degrees over the time course of the experiment.

### Effect of inorganic nutrient amendment on bacterial community composition

**Figure 7:**
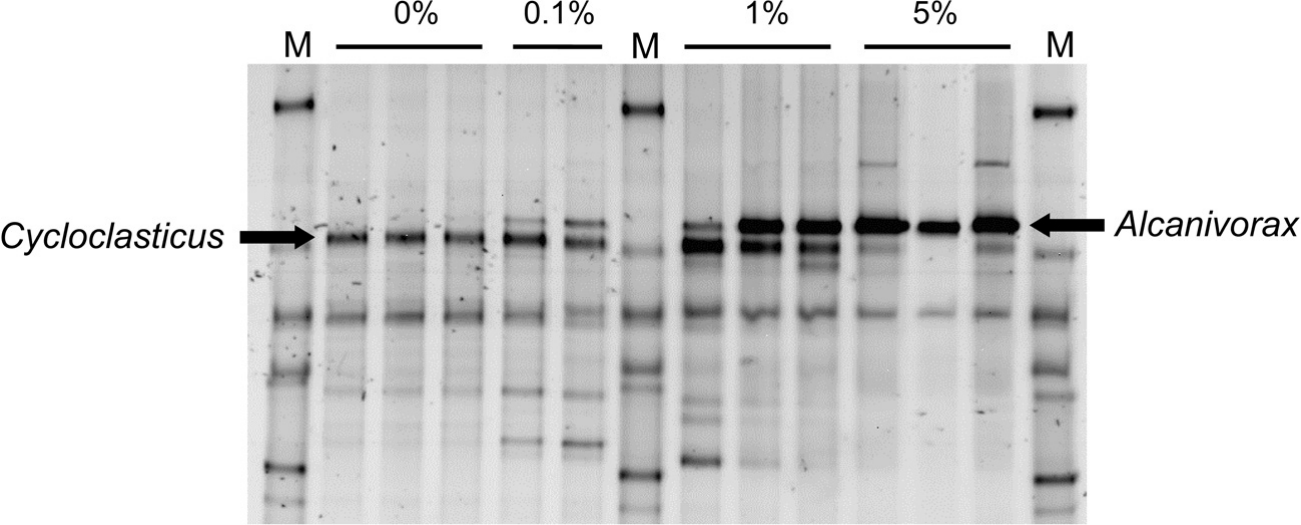
**Denaturing gradient gel electrophoresis of 16S rRNA gene fragments from crude oil and nutrient treated beach microcosms incubated for 6 days**. Fragments corresponding to *Cycloclasticus* spp and *Alcanivorax* spp, are indicated. All microcosms were treated with 0.5%P w/w of oil and the values above the lanes indicate the percentage of inorganic N added relative to the mass of oil.

Bacterial communities in microcosms treated with crude oil and different levels of inorganic nutrients were characterized by DGGE analysis of PCR-amplified 16S rRNA genes (Figure 7). Following 6 days of incubation a differential response in the bacterial communities to nutrient amendment was observed with bands corresponding to *Cycloclasticus* sp. being detected at lower nutrient concentrations (0–1% N w/w of oil) and *Alcanivorax* spp. becoming much more prevalent at higher nutrient concentrations (Figure 7). End-point PCR using *Alcanivorax* specific primers demonstrated that only 20% of samples (3 out of 15) treated with 0.8%N/0.5%P or less harbored detectable *Alcanivorax* whereas in samples treated with 1–5%N/0.5%P, 100% of samples (12 out of 12) harbored detectable *Alcanivorax* 16S rRNA genes. Interestingly all three microcosms treated with 0.5%N/0.5%P contained *Alcanivorax* 16S rRNA genes detectable by end-point PCR while no samples from 0.8%N/0.5% P-treated microcosms contained detectable *Alcanivorax* 16S rRNA genes. The selection of *Alcanivorax* at higher inorganic nutrient concentrations was consistent with the greater alkane degradation observed in these samples.

**Figure 8:**
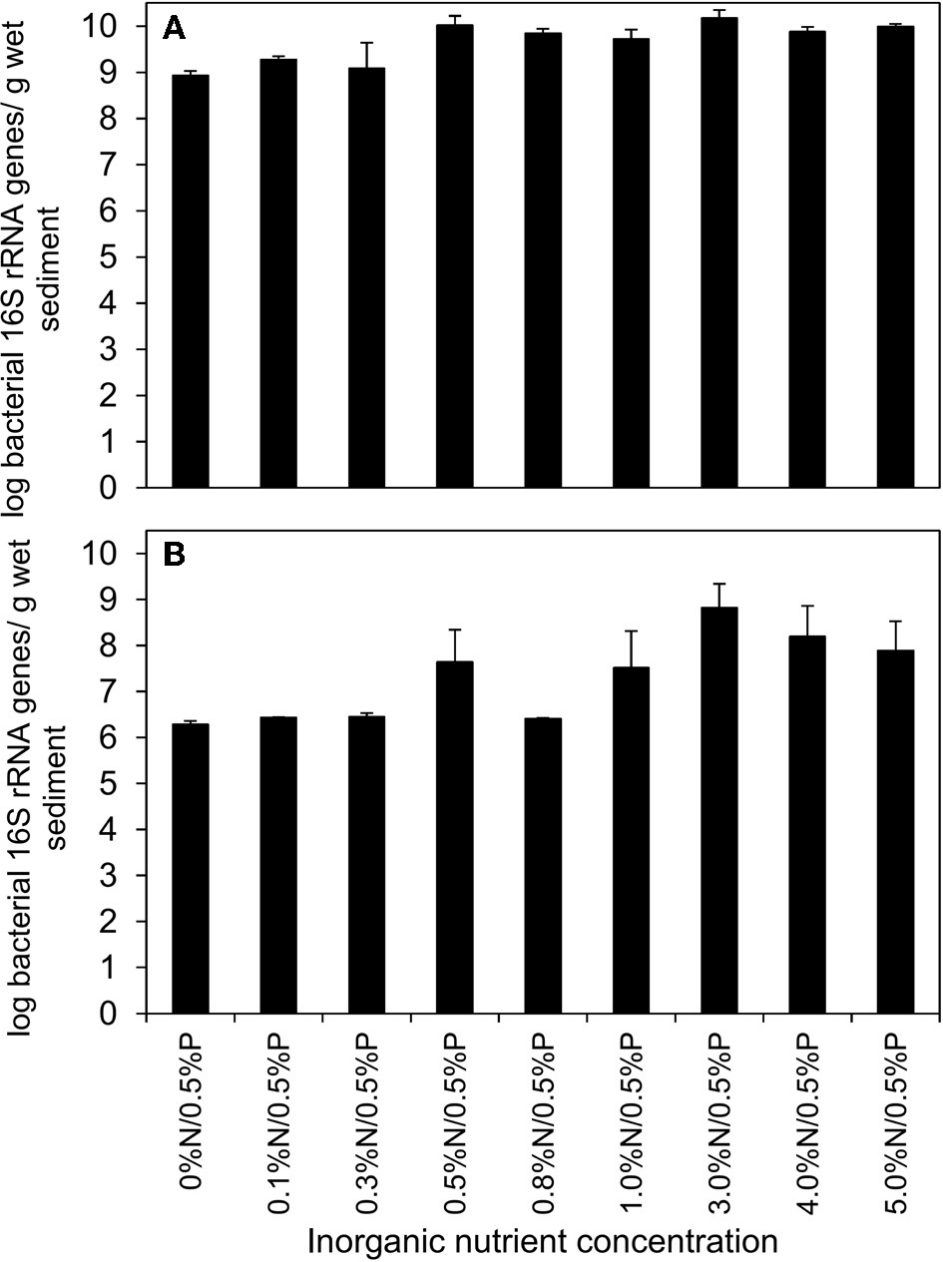
**qPCR analysis of total bacterial 16S rRNA genes (A) and *Alcanivorax* spp. 16S rRNA genes (B) from crude oil and nutrient treated beach microcosms incubated for 6 days**. All microcosms were treated with 0.5% P w/w of oil and the values below the columns indicate the percentage of inorganic N added relative to the mass of oil. Each data point represents average value of three replicates.

Quantification of bacterial 16S rRNA genes by qPCR showed a small, but significant increase in total bacterial abundance relative to nutrient levels (ANOVA: *P* = 0.008). The log bacterial gene abundance was 8.93 ± 0.09/g in sediments treated with 0%N/0.5%P with a maximum value of 10.17 ± 0.18/g sediment treated with 3%N/0.5%P (Figure 8A). The differences were due to higher bacterial 16S rRNA gene abundance in microcosms treated with nutrient concentrations greater than 0.3%N/0.5%P and at all nutrient treatments greater than this, there was no significant difference in total bacterial 16S rRNA gene abundance (ANOVA: *P* = 0.14).

Log 16S rRNA gene abundance determined using *Alcanivorax* specific primers ranged from 6.28 ± 0.08/g in sediments treated with 0%N/0.5% P to 8.82 ± 0.52/g, in sediments that received 3% N/0.5% P (Figure 8B). There were significant differences in *Alcanivorax* 16S rRNA gene abundance (ANOVA: *P* = 0.014). *Alcanivorax* 16S rRNA gene abundance in microcosms treated with 0.8% N/0.5% P had anomalously low 16S rRNA gene abundance (log abundance, 6.41 ± 0.02/g sediment) and excluding this value which was not significantly different from *Alcanivorax* 16S rRNA gene abundance at all nutrient concentrations less than 0.5% N/0.5% P (ANOVA: *P* = 0.275), showed that at all other nutrient concentrations greater than 0.3% N/0.5% P *Alcanivorax* genes were significantly more abundant than at lower nutrient levels, while there was no difference in abundance in all treatments greater than 0.3% N/0.5% P (ANOVA: *P* = 0.671).

## Discussion

### Kinetics of inorganic nutrient-stimulated crude oil biodegradation

Biostimulation with N and P is an effective method for enhancing the rate of oil bioremediation (Atlas and Bartha, 1972, 1973; Bragg et al., 1994; Venosa et al., 1996; Röling et al., 2002; McKew et al., 2007b; Coulon et al., 2007). Early studies of Atlas and Bartha (1972) were the first to demonstrate inorganic N and P-mediated stimulation of crude oil biodegradation and that both N and P amendment were required for oil degradation. Subsequently the feasibility of using oleophilic fertilizer as biostimulating agents was demonstrated (Atlas and Bartha, 1973). The enhancement of hydrocarbon degradation rate with increasing nitrate concentration (Boufadel et al., 1999), and nitrate plus sand amendments (Beolchini et al., 2010) has also been demonstrated. In the study of Beolchini et al. (2010) the sand enhanced biodegradation of high molecular weight aliphatic hydrocarbons and it was suggested that this was effective because it increased the surface area of the solid/liquid interface in the sediment and increased oxygen diffusion and mass transfer. However, these studies did not attempt to systematically analyze the nutrient enhanced kinetic parameters of crude oil biodegradation. Therefore, the focus of the present study was to evaluate the kinetic parameters for crude oil degradation in relation to N and P treatments.

Oil-stimulated CO_2_ production rate was used as a proxy for hydrocarbon degradation to determine initial rates in relation to nutrient levels and oil concentration to estimate kinetic parameters that may be useful for modeling crude oil bioremediation programmes for marine beach sediments. N amendment with no added P led to a small but significant stimulation of oil degradation (Figure 1), however when P-limitation was alleviated there was a greater enhancement in the rate of oil degradation up to around 0.8 to 1% N w/w of oil (Figure 1). While q_max_ values were four-fold higher when both N and P limitation were alleviated compared to alleviation of N limitation alone, K_s_ values for N were similar irrespective of P provision (0.72 ± 0.32 μmol N/g sediment and 1.99 ± 0.87 μmol N/g wet sediment). The water content of the sediments was determined to be 25.16 ± 0.09% (*n* = 3) and 250 μl of nutrient solution was added to each sample (i.e., water content of 2.766 ml per microcosm) and on this basis the K_s_ values were converted to molar concentrations. This gave a Ks value of 2.60 ± 1.16 mM with no P amendment and 7.19 ± 3.14 mM with N and P amendment. This is several orders of magnitude higher than the range of 6.9–122.4 μM reported for heterotrophic bacteria (Reay et al., 1999) and may reflect the fact that the key alkane degraders in the microcosms are *Alcanivorax* sp. which are known to be stimulated during bioremediation treatments and thus may be better adapted to relatively high inorganic nutrient concentrations. The K_s_ value for P was much lower at 63 ± 95 nmol/g wet sediment which translates to 227.77 ± 343.46 μM. The mean value obtained is much (orders of magnitude) higher than K_s_ values typically reported for phosphate utilization by bacteria and aquatic microbial communities which are usually sub micromolar (Vadstein and Olsen, 1989, 0.013–0.247 μM; Schowanek and Verstraete, 1990, 0.17 μM; Cotner and Wetzel, 1992, 0.019–0.225 μM). This may reflect adaptation of these specialist hydrocarbon-degrading taxa to high nutrient concentrations typical of conditions that are used to promote hydrocarbon bioremediation. Many pure cultures of *Alcanivorax* spp. are available and kinetic analysis with respect to N and P utilization would be highly informative in this regard. It should however, be noted that the error on the estimate of K_s_ for phosphate is large (±151%) and indicates that there is a statistical probability that K_s_ has a negative value. This is clearly not possible and one would have to conclude that the lower bound must be a small non-zero value. Putting this statistical incongruity to one side the important point is that it is difficult to draw reliable conclusions about the specific kinetic characteristics of the hydrocarbon degrading organisms with respect to inorganic phosphate from these data. Future studies should focus on analysing the response of hydrocarbon degraders to P at sub micromolar levels.

The occurrence of high affinity permeases for inorganic N and P in the genome of *Alcanivorax borkumensis* SK2 (Schneiker et al., 2006) seems to contradict the findings of the present study, however, as far as we are aware these have been annotated largely on the basis of sequences from known permeases and there is no direct evidence available regarding the kinetic of these permeases. Moreover organisms may have different sets of permeases with different affinity for inorganic nutrients allowing them to adapt rapidly to a change from nutrient limited to nutrient replete conditions. However, such conclusions would need, to be supported by detailed proteomic analysis of the response of *Alcanivorax* to growth at different nutrient levels (Sabirova et al., 2006).

### Kinetic response of crude oil degrading microbial communities to oil loading

Microbial activity generally increases in proportion to an accessible carbon and energy source. However, high concentrations of hydrocarbons have been shown to inhibit oil biodegradation either by causing nutrient or oxygen limitation or through direct toxicity of volatile hydrocarbons (Fusey and Oudot, 1984; Leahy and Colwell, 1990). Therefore, in addition to assessing the kinetics of hydrocarbon degradation in relation to inorganic nutrient availability we determined the effect of sediment oil loading on biodegradation rates when N and P were not limiting. CO_2_ evolution rates increased with increasing oil content from 1 to 50 mg crude oil/g sediment (5% oil by weight; Figure 3). This encompasses the range of contamination levels observed following the Exxon Valdez Spill in Prince William Sound, Alaska. The levels of oil in surface sediments in Prince William Sound were highly variable with an average value of 12.2 ± 18.6 mg/g sediment (Bragg et al., 1992). This suggests that effective biodegradation of the labile components of crude oil is likely achievable at oiling levels typically seen in oil spill-affected sediments in the field. Nevertheless, much higher levels of oil contamination (up to 510 mg/g) have been reported following the Deepwater Horizon blow out (Lin and Mendelssohn, 2012). The marsh sediments studied by Lin and Mendelssohn (2012) were from Bay Jimmy within Barataria Bay. Sediments in Barataria bay range from fine sand to coarse silts (most sediment particles in the range 2–5 in the phi scale of Krumbein and Aberdeen, 1937) and Bay Jimmy sediments specifically are organic rich (4.0–16.2% TOC by weight of sediment; Natter et al., 2012). This suggests that oxygen depletion in these sediments may contribute to the relative persistence in these sediments and indeed evidence has been presented that suggests that oiling of these sediments promoted sulfate-reduction (Natter et al., 2012).

The half saturation constant for crude oil in our sediments was estimated to be 5.83 ± 1.46 mg oil/g wet sediment. While this provides a practically useful K_s_ value for modeling the kinetics of field scale bioremediation it is not straightforward to compare this with literature values for pure compound and/or pure cultures of microorganisms due to the complexity of the mixture of carbon sources and their low water solubility. If the oil masses are converted to moles of carbon (crude oil is typically 84% carbon by weight) this translates into a K_s_ value of 388 ± 97 μmol C/g sediment. We know from oil chemistry data that only a fraction of the alkanes (*n*C_13_–*n*C_17_) were being degraded in the system and in the freshly added oil the mass of *n*C_13_–*n*C_17_ was equivalent to about 3% of the total oil mass, giving a K_s_ value for the alkanes which were actually being degraded of 11.64 μmol C/g sediment. The low aqueous solubility of alkanes also needs to be considered in this context (e.g., 1.7 nM for hexadecane in seawater at 25°C; Verschueren, 1983). Moreover, aqueous solubility defined under standard physical and chemical conditions may not be truly representative of the situation *in situ*, where biosurfactants may substantially increase the effective aqueous solubility. Nevertheless, the K_s_ values reported in terms of mass of sediment would be equivalent to orders of magnitude greater than K_s_ values reported for hydrocarbon degradation by pure cultures of bacteria which are in the micromolar or even sub-micromolar range. The toluene degrader *Cycloclasticus oligotrophicus* for example has the lowest known K_s_ for an organic substrate (0.014 μM; Button et al., 1998).

While the kinetic parameters we have estimated will be useful for modeling the fate of the more labile components of crude oil, as these become degraded more persistent fractions of the oil will be degraded more slowly. To address this, studies of partially degraded and heavier oils will be required. Interestingly, it has been reported that nominally labile crude oil hydrocarbons have persisted in subsurface sediments from Prince William Sound, Alaska, even 16 years after the *Exxon Valdez* oil spill (Short et al., 2007). This persistence could be explained by anoxia reducing the rates of hydrocarbon degradation, though this was discounted by Short et al. (2007) due to high levels of tidal flushing in the sediments. It was however suggested that nutrient availability may have been a factor in the persistence of these hydrocarbons, and perhaps more importantly the formation of water in oil emulsions (mousse), which reduce the surface area of oil available for microbial attack (Short et al., 2007). Subsequent studies however demonstrated that the long term residual oil was biodegradable and that inorganic nutrients and oxygen stimulated its biodegradation (Venosa et al., 2010). These studies emphasize the need to consider more than just microbiological factors when assessing the fate of spilled oil in the field.

### Suppression of bioremediation by high levels of inorganic nutrients

Recommendations for nutrient levels required for crude oil bioremediation are often given in terms of the mass of nutrients required relative to the mass of oil (Röling et al., 2004). Moreover arguments surrounding addition of excessive amounts of nutrients normally relate to avoiding eutrophication of neighboring water bodies (Swannell et al., 1996; Röling et al., 2004). For this reason we determined the response of oil degrading microorganisms to increasing levels of oil in the presence of a constant proportion, but increasing absolute amounts of nutrients. In experiments with increasing oil concentration above 12.5 mg oil/g sediment, marked inhibition of oil degradation was noted (Figure 4). This was shown to be a consequence of toxicity of high levels of nutrients rather than an effect of higher levels of oil (Figure 5). Converting the added nutrients into an aqueous concentration based on the water content of the sediments indicated that inhibition of oil degradation occurred at sodium nitrate and potassium orthophosphate concentrations of 238 mM nitrate and 10.8 mM phosphate or greater. These absolute concentrations are very high (almost 3 orders of magnitude greater than the K_s_ values determined here), but serve to underline the importance of designing treatment strategies on more than a simple mass balance of oil carbon relative to inorganic nutrient levels. It is possible that a similar effect might result from oxygen depletion at higher oil loadings however in the sediment containing 50 mg oil/g of sediment and 5%/0.5% N/P by mass, oxygen was depleted by only 24.0 ± 2.8% over the incubation period relative to oxygen levels at the start of the experiment. This compares with the treatment with 12.5 mg oil/g of sediment and 5%/0.5% N/P by mass where oxygen was depleted by 31.3 ± 2.8% from the levels at the start of the experiment. Moreover inhibition of oil degradation at 50 mg oil/g of sediment did not occur when lower absolute concentrations of nutrients were provided (Figure 3).

### Alkane degradation and growth of *Alcanivorax* in short term incubations

Biodegradation of saturated and aromatic hydrocarbons in crude oil contaminated environments has been previously demonstrated (Kasai et al., 2002a,b; Röling et al., 2002; Singh et al., 2011). The lack of detectable degradation of total resolved alkanes in our experiments is consistent with the short incubation period of the experiments which were designed to determine initial rates of hydrocarbon degradation, not the full extent of degradation possible. Similar experiments conducted over much longer timescales (30–90 days) typically show complete removal of the resolved alkanes. More detailed analysis of hydrocarbon degradation based on ratios of *n*-alkanes to pristane demonstrated that there was modest degradation of lower molecular weight alkanes with increasing inorganic nutrient concentrations (Figure 6). This is consistent with the increased rates of CO_2_ production and increase in abundance of *Alcanivorax* observed at higher nutrient levels (Figures 1, 8)

*Alcanivorax* spp. have been found to be globally significant for *in situ* degradation of straight and branched chain alkanes in marine environments (Dyksterhouse et al., 1995; Yakimov et al., 2005; Head et al., 2006; McKew et al., 2007a,b). A greater decrease in the ratio of *n*C_13_: pristane as compared to *n*C_17_: pristane and *n*C_25_: pristane ratios suggest a preference for degradation of low molecular weight alkanes by the *Alcanivorax* sp. detected in our experiments.

It is also interesting to note that at low nutrient concentrations *Cycloclasticus*-like bacteria were prevalent. These are known aromatic hydrocarbon-degrading bacteria with particularly high substrate affinities which contrasts with *Alcanivorax* which was strongly selected at higher nutrient concentrations (Figure 7) consistent with the high K_s_ values determined in these experiments. Interestingly despite the significance of inorganic nutrient provision for stimulation of crude oil biodegradation, and the fact that this has been known for several decades, there are relatively few data in the literature on the kinetic parameters for oil degradation with respect to inorganic N or P. This is an important gap in our knowledge of the ecology of hydrocarbon-degrading bacteria and has practical implications for understanding the fate of crude oil in the environment. The present day focus on omics- enabled studies of hydrocarbon-degrading communities would be perfectly complemented by more studies to determine fundamental kinetic and physiological properties of both pure cultures and natural communities of hydrocarbon-degrading bacteria. It will be interesting to note if the high K_s_/low affinity kinetics noted in this study are reflected in the kinetic properties of pure cultures and how they are affected by environmental conditions such as temperature. This kind of information will be essential for incorporation of ecological principles such as resource ratio theory into modeling approaches to better understand the fate of spilled oil (Smith et al., 1998). Both theoretical and experimental approaches to understanding the competitiveness of different hydrocarbon-degrading bacteria under different scenarios would be facilitated by such basic knowledge of the physiology of hydrocarbon degrading bacteria (McKew et al., 2007b). This is an attractive proposition given that many of the key players in marine hydrocarbon degradation are available in culture (Yakimov et al., 2007). In addition genome sequences have been determined for a number of these taxa and it is only a matter of time before many more marine obligate hydrocarbon degrading bacterial genomes are sequenced (Schneiker et al., 2006).

## Conclusions

The data presented here provide a systematic assessment of key factors that control the biodegradation of crude oil in beach sediments and provide kinetic parameters that can be used in kinetic modeling of beach oil spill bioremediation. Our results not only confirm that crude oil biodegradation in marine beach sediments is sensitive to the level of inorganic N and P nutrient treatments but that the maximum rates of crude oil biodegradation achievable are approximately 16 μmol C/g sediment/day at the incubation temperature of our experiments (24°C). Half saturation constants for N and P in the form of nitrate and phosphate are high compared to values typically seen in pure cultures of heterotrophic bacteria and underline the importance of maintaining high inorganic nutrient concentrations to accelerate hydrocarbon degradation. The half saturation constants also provide fundamental parameters for future kinetic modeling of bioremediation of beached oil spills. The high nutrient levels that promote crude oil biodegradation also select for specialized alkane degrading bacteria from the genus *Alcanivorax* while selecting against aromatic hydrocarbon degrading bacteria such as *Cycloclasticus* sp. This suggest that by manipulation of nutrient amendments it may be possible to balance aromatic and aliphatic hydrocarbon degradation, albeit at the expense of lower overall rates of biodegradation due to the lower levels of nutrients required to stimulate aromatic hydrocarbon degrading *Cycloclasticus* spp. We also demonstrated that nutrient-enhanced bioremediation is effective only up to a point and care should be taken in oil spill bioremediation, not only to protect against eutrophication by avoiding excessive nutrient loading, but also to avoid inhibition of hydrocarbon degradation at high nutrient levels. Similar analyses on a wider range of sediments will establish if the observations we have made in one beach sediment apply broadly across a range of environments and geographical locations.

## Author contributions

Ian M. Head, Arvind K. Singh, and Neil D. Gray developed the concept and designed experiments; Arvind K. Singh prepared and analyzed microcosm experiments. Arvind K. Singh, Neil D. Gray, and Angela Sherry conducted headspace gas analysis and microbial community analysis. Arvind K. Singh, Bernard F. J. Bowler, and D. Martin Jones were responsible for and conducted crude oil analysis. Data analysis and interpretation was conducted by Arvind K. Singh, Ian M. Head, Angela Sherry, and Neil D. Gray, Arvind K. Singh, and Ian M. Head wrote the manuscript with critical input from Angela Sherry, Neil D. Gray, and D. Martin Jones.

### Conflict of interest statement

The authors declare that the research was conducted in the absence of any commercial or financial relationships that could be construed as a potential conflict of interest.

## Supplementary material

Figure S1 **Cumulative CO_2_ production in beach sediment microcosms**. Data from microcosms treated with crude oil plus inorganic nitrogen and phosphorus (black circles), inorganic nitrogen and phosphorus but no oil (gray circles) and killed control microcosms treated with formaldehyde (2% final concentration) and crude oil plus inorganic nitrogen and phosphorus (white circles). Oil treated microcosms contained 10 mg crude oil/g sediment and inorganic nutrient amendments equated to 5% N and 0.5% P by weight of the oil.

Figure S2 **Effect of headspace flushing on physical removal of volatile saturated hydrocarbons from beach sediment microcosms**. Concentrations of the individual compounds (C) after flushing the headspace with different volumes of air are presented relative to initial headspace concentration (C_0_) of the volatile hydrocarbons.
