# Supplementary material for: Kinetic parameters for nutrient enhanced crude oil biodegradation in intertidal marine sediments
Source: Front Microbiol. 2014 Apr 11;5:160. doi: 10.3389/fmicb.2014.00160 (PMC3990054; doi:10.3389/fmicb.2014.00160)
Supplement: Figure S1 — Cumulative CO2 production in beach sediment microcosms. Data from microcosms treated with crude oil plus inorganic nitrogen and phosphorus (black circles), inorganic nitrogen and phosphorus but no oil (gray circles) and killed control microcosms treated with formaldehyde (2% final concentration) and crude oil plus inorganic nitrogen and phosphorus (white circles). Oil treated microcosms contained 10 mg crude oil/g sediment and inorganic nutrient amendments equated to 5% N and 0.5% P by weight of the oil. [file Presentation1.PDF]

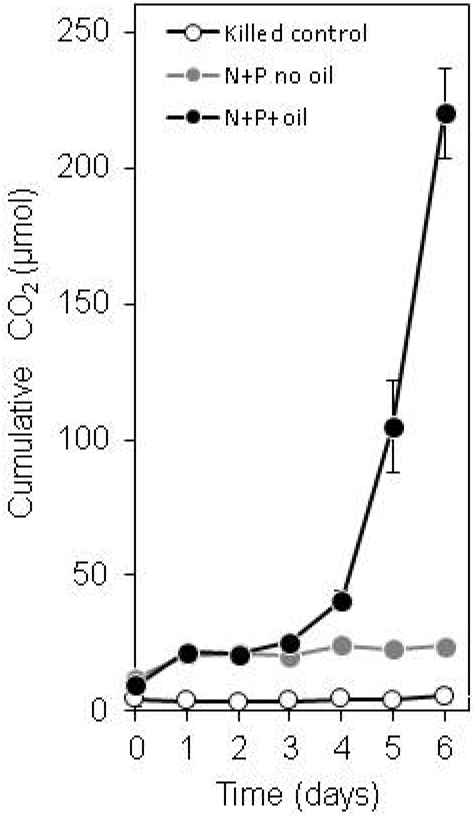

Fig S1: Cumulative CO_2 production in beach sediment microcosms. Data from microcosms treated with crude oil plus inorganic nitrogen and phosphorus (black circles), inorganic nitrogen and phosphorus but no oil (gray circles) and killed control microcosms treated with formaldehyde (2% final concentration) and crude oil plus inorganic nitrogen and phosphorus (white circles). Oil treated microcosms contained 10 mg crude oil/g sediment and inorganic nutrient amendments equated to 5%N and 0.5%P by weight of the oil.
