# Supplementary material for: Kinetic parameters for nutrient enhanced crude oil biodegradation in intertidal marine sediments
Source: Front Microbiol. 2014 Apr 11;5:160. doi: 10.3389/fmicb.2014.00160 (PMC3990054; doi:10.3389/fmicb.2014.00160)
Supplement: Figure S2 — Effect of headspace flushing on physical removal of volatile saturated hydrocarbons from beach sediment microcosms. Concentrations of the individual compounds (C) after flushing the headspace with different volumes of air are presented relative to initial headspace concentration (C0) of the volatile hydrocarbons. [file Presentation2.PDF]

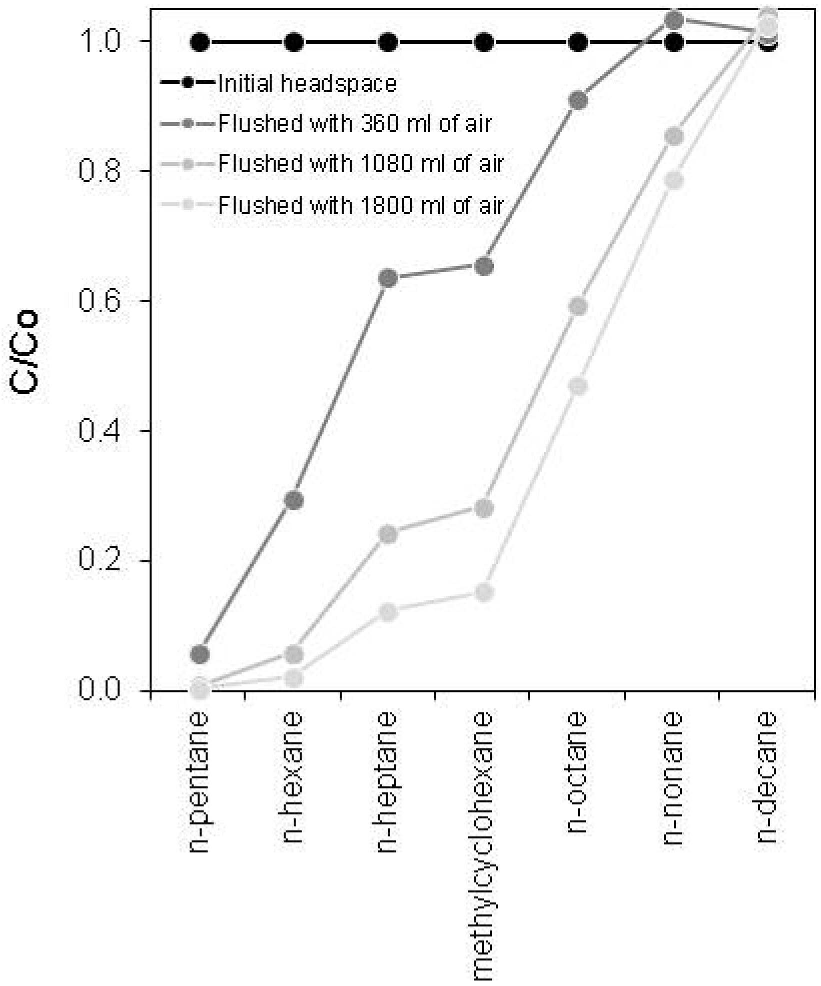

Fig S2: Effect of headspace flushing on physical removal of volatile saturated hydrocarbons from beach sediment microcosms. Concentrations of the individual compounds (C) after flushing the headspace with different volumes of air are presented relative to initial headspace concentration (C_0) of the volatile hydrocarbons.
